# Spatial Inequality in Under‐Five Children Undernutrition: Evidence From Ethiopia

**DOI:** 10.1155/jotm/7022986

**Published:** 2026-06-26

**Authors:** Abigail Chari, Tafadzwa Dzinamarira, Patrick Gad Iradukunda, Elliot Mbunge, John Batani, Godfrey Musuka

**Affiliations:** ^1^ Research on Socioeconomic Policy, Department of Economics, Stellenbosch University, Stellenbosch, South Africa, sun.ac.za; ^2^ School of Health Systems and Public Health, University of Pretoria, Pretoria, South Africa, up.ac.za; ^3^ Rwanda Food and Drug Authority, Kigali, Rwanda; ^4^ University of Johannesburg, Johannesburg, South Africa, uj.ac.za; ^5^ Botho University, Maseru, Lesotho, bothouniversity.com; ^6^ International Initiative for Impact Evaluation, Harare, Zimbabwe

**Keywords:** child undernutrition, Ethiopia, spatial inequality, stunting, underweight, wasting

## Abstract

Many developing countries are overburdened by child undernutrition, skewed towards poor and rural populations. This has long‐lasting effects on adult livelihoods through reduced productivity and living standards, which vary across different countries and within countries. In that regard, we examined the spatial disparities in child undernutrition and its contributors in Ethiopia. Using 2019 Ethiopia Demographic Health Survey data and subnational boundaries as well as thematic maps and spatial autoregressive model at the cluster or kebele level, we found the presence of spatial disparities in child undernutrition, where children in the northern and northeast kebeles are more likely to have undernutrition than other kebeles. In addition, we found that aridity, precipitation, skilled health worker availability, geographical location and women’s education significantly influence undernutrition. We also found positive and significant spillover effects in stunting, wasting and undernutrition between clusters, showing spatial dependence in Ethiopia. There is a need to focus on underserved regions to ensure that resources are allocated to those in need. Policymakers need to design better educational and economic strategies to increase food and nutrition security for children from poor and rural households, especially those in southern, northern, northeast and some eastern regions. Thus, there is a need to ensure targeted nutrition programmes in arid regions, climate‐resilient agricultural interventions, expansion of maternal education programmes and strengthening rural health systems to improve nutrition outcomes for children under five.

## 1. Introduction

Child undernutrition is prevalent in developing countries, primarily affecting poor and rural populations [1, 2]. More specifically, children under five are more susceptible to undernutrition, in the form of stunting, wasting and underweight, than other age groups. As clearly indicated by the World Health Organization [3], one in every five children under five was stunted, while one in every ten was wasted in 2019. This worsens the already existing infant and under‐five mortality, where around half of the under‐five mortality is linked to child undernutrition. Undernutrition remains a burden to vulnerable groups of people in society, contributing towards high healthcare costs, poor cognitive development and poor educational outcomes. In the long term, undernutrition leads to poor health outcomes, reduced productivity and a vicious cycle of poverty [4, 5], hence economic and developmental effects.

Similarly, Ethiopia has a high prevalence of undernutrition. Regardless of the high prevalence of overall undernutrition, stunting declined from 51% in 2005 to 37% in 2019, indicating a 27.5% decrease in stunting [6]. As noted by the Ethiopian Public Health Institute and ICF [6], two in every five under‐five children were short for their age, one in every twenty was thin for their height, and one in every five was thin for their age in 2019. Children residing in urban areas (15%) were less likely to be stunted than those from rural areas (23%) in 2019 [6]. Factors affecting child undernutrition include, but are not limited to, child’s gender and age, household wealth, food security and geographical location and mother’s characteristics (education, marital status and exposure to media) [7–9]. UNICEF [10]’s framework noted several factors that affect undernutrition, including diets, care, food security, a healthy environment, and human, economic and financial resources. Although child undernutrition is high nationwide, regional disparities in undernutrition exist within a country. In that regard, children from Afar, Amhara, Benishangul‐Gumuz and Tigray had the highest prevalence of stunting, while Addis Ababa, Gambela and Dire Dawa regions had the lowest stunting in 2019 [6].

Previous literature noted that demographic, social and economic variables affect malnutrition in developing countries [2, 11–14]. Chari [5] explored spatial disparities in child undernutrition in Zimbabwe. Specific to Ethiopia, a few studies have examined factors affecting undernutrition as well as spatial variations in stunting [15, 16], with limited literature on spatial inequality in child undernutrition. This is regardless of the need to reveal these inequalities, which are important in understanding underserved regions in Ethiopia. Several studies in Ethiopia focused on spatial factors influencing undernutrition, using logistic regression models [15–18], Bayesian geostatistical analysis [19] and the concentration index [20]. These studies have not used spatial regression models, which are important to determine the dependencies and spillovers between areas. Thus, this paper bridges the existing gap in literature by examining spatial disparities and cluster‐level factors that affect undernutrition among children under five in Ethiopia using a spatial autoregressive (SAR) model. To achieve this objective, we use data from the 2019 Ethiopia Demographic Health Survey (DHS), focusing on children under five. Although several studies have been conducted on regional analyses of undernutrition in Ethiopia [15–17, 21], a few studies, including Haile et al. [18], have been conducted using the lowest administration level, ward or kebele level. We, therefore, showed the spatial disparities in child undernutrition using the lowest administrative data (kebeles or ward‐level), which remains unexplored in the literature.

Ward‐level or cluster‐level analysis captures the localised disparities in environmental and socioeconomic conditions while reducing aggregation bias and ensuring that the model is more accurate. Identifying cluster‐ or ward‐level disparities in child undernutrition is important in making informed decisions by allocating resources to underserved wards in the country. In this analysis, we found evidence of spatial dependence in undernutrition in Ethiopia for effective and efficient allocation of resources. Moreover, understanding these inequalities not only helps in reducing stunting, wasting and underweight but also has long‐term effects on education, labour market outcomes and income inequality. This is important in ensuring the attainment of Sustainable Development Goals (SDGs) 2 (zero hunger) and 3 (good health and well‐being).

## 2. Methods

### 2.1. Data

We use the EDHS to examine the factors affecting child undernutrition in Ethiopia. This survey was conducted by the Ethiopian Public Health Institute, together with other stakeholders who provided technical and financial assistance. The survey collects information on household characteristics, child health outcomes, child feeding practices and healthcare access, to mention a few. The EDHS is conducted nationwide with representation from all urban and rural areas within the country’s regions. The data are based on a two‐stage sampling method, where the selection of enumeration areas is done first and then the assignment of households to their respective enumeration. EDHS collects data using questionnaires for men, women, children aged 5–17 and children under five. We used the children’s questionnaire, which contained relevant information for analysis.

We use the 2019 EDHS based on information from the 2019 Ethiopia Population and Housing Census [6]. The 2019 EDHS was the first minisurvey, but the fifth DHS in the country, with data collected from March to June 2019. The households within the enumeration areas were randomly selected. Of the 8794 households selected during the survey, 8663 were interviewed yielding a 99% success rate [6]. The analysis is conducted for children under five, including mothers’ and household information, where some of the variables had missing observations. The unit of measurement for this analysis was the enumeration areas in Ethiopia. We also use data from the 2019 DHS geospatial covariate datasets, which comprises information on climate factors in the analysis. The climate variables extracted from this dataset include rainfall, temperature, aridity and length of the growing season.

We extract the third Ethiopian subnational administrative boundaries from Human Data Exchange [22] to merge the GPS from these data to kebeles (wards) from the EDHS. The EDHS‐masked clusters are linked to the kebeles in the subnational administrative boundaries, following Chari [5] and Wilson et al. [23]. Matching EDHS and the subnational administrative boundaries effectively identifies the third administrative unit in both datasets, which is important for analysis [5, 23]. EDHS cluster coordinates from urban areas were displaced up to 2 km, while those from rural areas were displaced up to 10 km to protect the individual’s anonymity [24]. The clusters were displaced while ensuring no overlaps of clusters between administrative areas, which might help in reducing biased estimates from the displacement.

The paper uses anonymised publicly available secondary data from the EDHS, accessed from the DHS programme with permission. When collecting information from the participants, DHS obtained ethical approval, and no further approvals are required for individual data analysis.

### 2.2. Definition of Variables

We use child undernutrition as the outcome variable, which is measured as the multidimensional variable of stunting, wasting and underweight. Stunting measures the height for the age of children under five, while wasting measures how thin the children are for their height [25, 26]. In addition, underweight measures the weight for age for children under 5 years. Stunting, wasting and being underweight are defined as z‐scores below negative two (−2) standard deviations. These are the outcome variables measured as the proportions of children stunted, wasted or underweight within a cluster in the analysis, respectively. Additionally, the multidimensional undernutrition shows the share of either stunted, wasted or underweight children in a cluster.

Several independent variables are used in this analysis: precipitation, aridity, number of wet days, daytime land surface, skilled health worker availability, geographical location and women’s education. Climate factors affect child undernutrition through their effect on food production, security and diversity. Adverse climates have a negative effect on food diversity, which compromises children’s nutritional status. The day land surface temperature is measured in degrees Celsius. Geographical location is crucial in determining child nutrition. Children from rural areas are more likely to be stunted, wasted and underweight than those from urban areas, owing to the less availability and accessibility of prenatal and postnatal care and information on nutritional outcomes. This is a binary variable where rural areas are assigned a value of one and zero for urban areas. We included the occupational and economic factors as they are part of a cluster development, considering that the analysis is not at the household level.

According to UNICEF [10], good care is also a determinant of nutrition, which is proxied by the availability of skilled health workers in this analysis, defined as the share of women who delivered at a health facility with the help of a health professional. As emphasised by Ntenda and Chuang [26], a considerable proportion of children with undernutrition reside in neighbourhoods with poor socioeconomic conditions and limited access to basic services. In addition, educated women are better informed about the significance of nutrition and effectively use the available resources to improve child nutrition [10, 27]. Women are largely responsible for their children’s well‐being, and their involvement in intra‐household decision‐making is important in alleviating child undernutrition [28]. Clustered women’s education is defined as the share of women with secondary education and above in a cluster.

## 3. Estimation Technique

### 3.1. Thematic Maps

We first used thematic maps to determine spatial disparities in undernutrition among children under five in Ethiopia. Thematic maps facilitate visual mapping, which enables the identification of the kebeles (wards) with a high prevalence of undernutrition in Ethiopia, showing geographical inequalities in stunting, wasting and underweight. This is important as decision‐makers use this information to make evidence‐based decisions, considering regional needs. The analysis also enables the decision‐makers to identify demographic commonalities by concentrating efforts on areas with high prevalence of undernutrition in Ethiopia.

### 3.2. SAR Model

Following existing literature and data structure, we used SAR model methods to estimate the factors affecting child undernutrition in Ethiopia. The SAR model illustrates the interaction between the endogenous and exogenous variables, where the endogenous variable is spatially lagged. The model highlights relationships between the undernutrition in one cluster and neighbouring clusters, explicated through spatially lagged undernutrition [29]. The SAR model is identified as follows:

(1) undernutritioni=α+xiβ+ρWundernutritionj+δi+εij,

where undernutrition is the outcome variable (representing stunting, wasting, underweight and multidimensional undernutrition of under‐five children) and *x* is the matrix of control variables (skilled delivery availability, perception of distance to health facility, women’s education, geographical location and precipitation, number of wet days, daily land surface temperature and aridity). *i* is the focal cluster, *j* is the cluster, while *ε*_*i* *j*_ denotes an error term. *β*_*i*_ shows the direction and magnitude of control variables that determine undernutrition among children under five in Ethiopia. *W*_*i* *j*_ is a contiguity spatial weighting matrix (*N*∗*N* matrix), while ρ is the coefficient for the spatial dependence.

The spatial weighting matrix measures the spillover effects of different variables and accounts for the differences in effects in areas. This shows that areas that are close to each other have more weight than those far from each other [30]. We use the contiguity rook weighting matrix that emphasises clusters that have the same boundaries to represent spillover effects [31]. In this regard, we first determine the validity of spatial panel data models using the Moran test and note that spatial models are appropriate. We then use the Lagrange multiplier to assess the validity of each spatial model, and the SAR model was noted to be the most appropriate for this analysis.

## 4. Results

Table 1 shows descriptive statistics. Over a third of the children in Ethiopia are short for their age, while around one‐fifth are too thin for their age. On average, 8.5% of the children are wasted. Most of the individuals in the survey reside in rural areas, with only a minority in urban areas (15.4%). In addition, Oromiya has a considerable proportion of individuals in the survey, followed by Southern Nations, Nationalities and People’s Region and, lastly, Dire Dawa. As indicated in Table 1, almost half of the individuals in the survey were attended by a skilled worker when they visited the health facilities. One in every 10 women has either secondary or/and tertiary education in Ethiopia.

**TABLE 1:** Descriptive statistics.

Variable	Obs	Mean	Std. Dev.	Min	Max
Stunting	690	0.336	0.169	0	0.750
Underweight	690	0.222	0.160	0	0.789
Wasting	690	0.085	0.109	0	0.615
Undernutrition	690	0.398	0.174	0	0.900
Precipitation	690	4.397	0.430	2.797	4.995
Skilled health worker availability	690	0.495	0.332	0	1
Aridity	690	3.012	0.568	0.901	3.746
Number of wet days	690	1.919	0.286	1.041	2.287
Daytime land surface	690	3.417	0.164	3.043	3.859
Women’s education	690	0.100	0.142	0	0.950
Location (ref: rural)	690	0.154	0.361	0	1
Regions					
Tigray	690	0.061	0.239	0	1
Afar	690	0.049	0.217	0	1
Amhara	690	0.172	0.378	0	1
Oromiya	690	0.310	0.463	0	1
Somali	690	0.081	0.273	0	1
Benishangul‐Gumuz	690	0.041	0.197	0	1
Southern Nations, Nationalities and People’s Region	690	0.200	0.400	0	1
Gambela	690	0.033	0.180	0	1
Harari	690	0.009	0.093	0	1
Addis Ababa	690	0.035	0.183	0	1
Dire Dawa	690	0.009	0.093	0	1

## 5. Regional Disparity by Kebeles (Wards)

**FIGURE 1:**
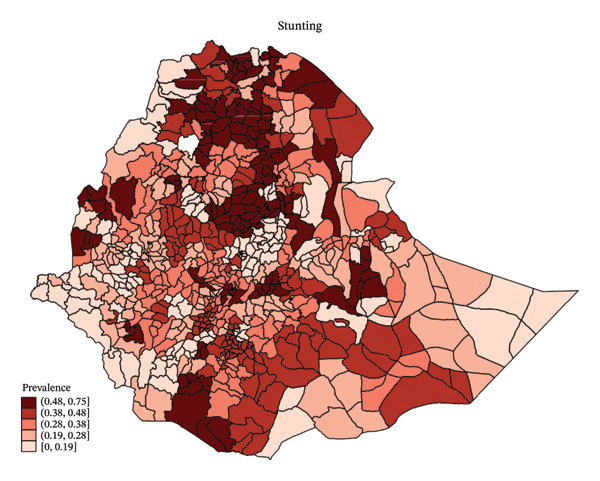
Regional disparities in stunting.

Figure 1 shows evidence of geographical differences in stunting at the kebeles level. Some kebeles in the northern, northeast, southwest, central and western regions have high prevalence of stunting. These include Tigray, Amhara, Afar and South Ethiopia. However, kebeles in eastern, central and western Ethiopia have a low proportion of stunted children. The Kebeles with a high prevalence of stunting are more than those with a low prevalence, showing that stunting prevalence is high in Ethiopia. Over half of the children in these kebeles were stunted compared to other kebeles in the country. These areas are primarily reliant on agricultural production and have a history of political and social complexities due to the diverse ethnic groups and conflicting interests in these areas.

**FIGURE 2:**
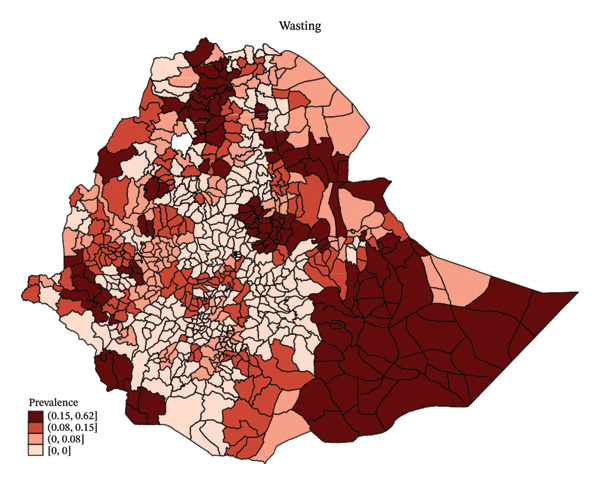
Regional disparities in underweight.

As indicated in Figure 2, regional disparities in underweight are evident in Ethiopia. Kebeles in the northern, northeast, southern and southeast regions have high stunting prevalence, while central, western and some parts of the southwest have a low underweight prevalence. Afar (Zones 1, 2, 3, 4 and 5 and Oromiya), Tigray (central Tigray, west Tigray and southern Tigray), parts of Somali (Alder, Gode and Warder), parts of Oromia (Borena Guji), Benishangul‐Gumuz (Tonga, Asosa and Kamashi) and South Wollo had a high prevalence of underweight children. Almost half of the kebeles in Ethiopia had over half of the child population who were considered underweight.

**FIGURE 3:**
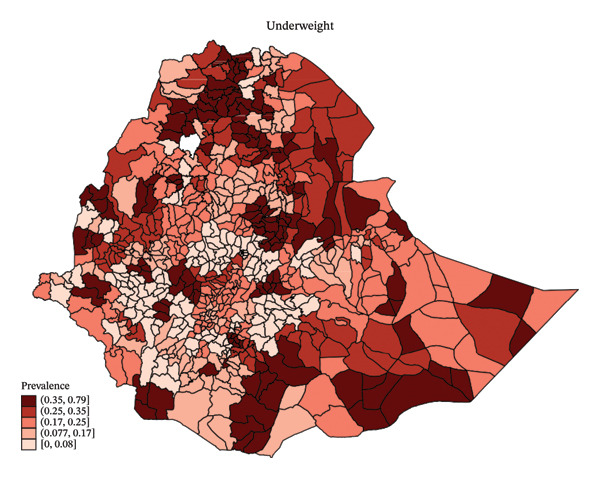
Regional disparities in wasting.

Figure 3 shows the regional disparities in wasting in Ethiopia. A considerable number of kebeles in the eastern and northern regions have a high prevalence of wasted children. These regions include Somali, Dire Dawa and some parts of Gambela, Afar, Tigray and Amhara. Central parts of Ethiopia have a low prevalence of wasting.

Northern, southern and northeast regions, particularly Tigray, Amhara and Afar, suffer a triple burden of stunting, underweight and wasting. At the same time, central and some western parts have a low prevalence of the three undernutrition indicators. Kebeles with wasted children are located in Warder, Liben, Alder, Gode, Korahe, Fik, Degehabour, Oromiya, parts of Shinille, Zone 1 (Afar), west Tigray, Zone 1 (Gambela) and South Omo. Compared to stunting and underweight, less than half of the kebeles have a high proportion of children who are too thin for their height in Ethiopia.

Table 2 shows the SAR model regression results using a contiguity weighting matrix. Models with region fixed effects and those without show that women’s education, the availability of skilled health workers and living in urban areas significantly reduce undernutrition in Ethiopia. This shows that higher maternal education and healthcare services are robust protective factors for undernutrition in the country. Daytime land surface temperature and aridity significantly increase stunting, while precipitation reduces stunting. However, precipitation increases wasting, while aridity decreases wasting and underweight in Ethiopia.

**TABLE 2:** Spatial autoregressive regression models.

Variables	(1)	(2)	(3)	(4)	(5)	(6)	(7)	(8)
	Stunting	Stunting	Wasting	Wasting	Underweight	Underweight	Undernutrition	Undernutrition
Precipitation	−0.332^∗∗^	−0.548^∗∗∗^	0.092	0.203^∗∗∗^	0.176	0.060	−0.366^∗∗∗^	−0.438^∗∗∗^
	(0.135)	(0.104)	(0.077)	(0.059)	(0.123)	(0.089)	(0.132)	(0.097)

Aridity	0.288^∗∗∗^	0.426^∗∗∗^	−0.121^∗^	−0.198^∗∗∗^	−0.226^∗∗^	−0.122	0.218^∗∗^	0.261^∗∗∗^
	(0.108)	(0.083)	(0.062)	(0.047)	(0.098)	(0.071)	(0.105)	(0.077)

Number of wet days	−0.017	−0.009	0.012	0.006	0.039	0.000	0.014	0.022
	(0.061)	(0.055)	(0.034)	(0.030)	(0.056)	(0.049)	(0.060)	(0.053)

Daytime land surface temperature	0.135^∗∗^	0.052	0.003	0.046	−0.029	0.043	0.007	−0.008
	(0.057)	(0.056)	(0.031)	(0.030)	(0.052)	(0.051)	(0.055)	(0.054)

Skilled health worker availability	−0.053^∗∗^	−0.010	−0.001	0.002	0.011	0.050^∗∗∗^	−0.009	0.040^∗^
	(0.021)	(0.021)	(0.012)	(0.011)	(0.019)	(0.019)	(0.020)	(0.020)

Women’s education	−0.298^∗∗∗^	−0.332^∗∗∗^	−0.070^∗∗^	−0.091^∗∗∗^	−0.330^∗∗∗^	−0.368^∗∗∗^	−0.389^∗∗∗^	−0.454^∗∗∗^
	(0.051)	(0.052)	(0.028)	(0.029)	(0.047)	(0.048)	(0.050)	(0.051)

Location (ref: rural)	−0.036^∗^	−0.073^∗∗∗^	−0.002	−0.001	−0.039^∗∗^	−0.054^∗∗∗^	−0.048^∗∗∗^	−0.083^∗∗∗^
	(0.019)	(0.019)	(0.010)	(0.010)	(0.017)	(0.017)	(0.018)	(0.018)

Constant	0.662^∗^	1.340^∗∗∗^	−0.054	−0.402^∗∗^	0.223	0.183	1.410^∗∗∗^	1.537^∗∗∗^
	(0.382)	(0.341)	(0.213)	(0.184)	(0.349)	(0.299)	(0.377)	(0.325)

*Weighted variables*								
*ρ*	0. 140^∗∗^	0.050	0.519^∗∗∗^	0.449^∗∗∗^	0.089	0.065	0.087^∗^	0.063
	(0.058)	(0.065)	(0.073)	(0.084)	(0.070)	(0.077)	(0.048)	(0.051)

Region FE	Yes	No	Yes	No	Yes	No	Yes	No
Observations	690	690	690	690	690	690	690	690

Standard errors in parentheses.

^∗∗∗^ *p* < 0.01.

^∗∗^ *p* < 0.05.

^∗^ *p* < 0.1.

The SAR coefficient (*ρ*) significantly and negatively influences stunting and wasting for under‐five children, showing that the localised spillover effects across neighbouring wards exist in Ethiopia. A unit increase in the average stunting in one region increases stunting in the other region by 0.14, while an increase in wasting in one region increases wasting in another by 0.52. This shows that stunting and wasting in one ward are determined by the conditions in another ward.

In addition, we also use the inverse distance weighting matrix to estimate the results as part of robustness checks, highlighted in Table 3. The inverse distance weighting was included to account for the distance decay and capture the broader environmental spillover effects that might not be shown using the contiguity‐based weights. Table 3 shows insignificantly different results from those in Table 4, except for the coefficients of the environmental factors, which show a more significant effect on undernutrition than under contiguity. This is important in noting that precipitation, aridity and temperature are not localised and undernutrition might be determined by the environmental factors from a distance. The contiguity specification is preferred as the main model as it produced more stable results, which are interpretable, while the inverse probability weighting is treated as a robustness check for the role of spatially continuous environmental factors. We also include the OLS regression results in Table 5 for robustness checks, which did not show much difference from the SAR results.

**TABLE 3:** SAR robustness checks using inverse distance weighting.

Variables	(1)	(2)	(3)	(4)	(5)	(6)	(7)	(8)
	Stunting	Stunting	Wasting	Wasting	Underweight	Underweight	Undernutrition	Undernutrition
Precipitation	−0.146	−0.524^∗∗∗^	0.469^∗∗∗^	0.551^∗∗∗^	0.394^∗∗∗^	0.087	0.012	−0.336^∗∗∗^
	(0.169)	(0.133)	(0.077)	(0.065)	(0.141)	(0.119)	(0.160)	(0.128)

Aridity	0.093	0.401^∗∗∗^	−0.520^∗∗∗^	−0.537^∗∗∗^	−0.451^∗∗∗^	−0.148	−0.167	0.164
	(0.147)	(0.113)	(0.067)	(0.056)	(0.121)	(0.103)	(0.138)	(0.108)

Number of wet days	0.037	−0.009	0.161^∗∗∗^	0.068	0.104^∗^	0.000	0.111^∗^	0.026
	(0.066)	(0.056)	(0.039)	(0.035)	(0.060)	(0.051)	(0.064)	(0.054)
Daytime land surface temperature	0.117^∗^	0.052	−0.031	0.042	−0.057	0.045	−0.043	−0.10
	(0.060)	(0.057)	(0.033)	(0.033)	(0.053)	(0.051)	(0.057)	(0.055)
Skilled health worker availability	−0.054^∗∗^	−0.009	−0.008	−0.000	0.012	0.052^∗∗∗^	−0.005	0.042^∗∗^
	(0.021)	(0.021)	(0.012)	(0.012)	(0.019)	(0.019)	(0.021)	(0.021)
Women’s education	−0.303^∗∗∗^	−0.340^∗∗∗^	−0.063^∗∗^	−0.097^∗∗∗^	−0.335^∗∗∗^	−0.378^∗∗∗^	−0.383^∗∗∗^	−0.467^∗∗∗^
	(0.052)	(0.052)	(0.030)	(0.030)	(0.047)	(0.047)	(0.050)	(0.050)
Location (ref: rural)	−0.036^∗^	−0.074^∗∗∗^	−0.003	−0.006	−0.039^∗∗^	−0.055^∗∗∗^	−0.050^∗∗∗^	0.086^∗∗∗^
	(0.019)	(0.019)	(0.011)	(0.011)	(0.017)	(0.017)	(0.018)	(0.018)

Constant	0.336	1.297^∗∗∗^	−0.832	−1.143^∗∗^	−0.186	0.139	0.738^∗^	1.341^∗∗∗^
	(0.429)	(0.384)	(0.230)	(0.208)	(0.377)	(0.350)	(0.411)	(0.371)

*Weighted variable*								
*ρ*	0. 395^∗∗^	0.075	3.373^∗∗∗^	2.328^∗∗∗^	0.676^∗∗∗^	0.063	0.575^∗∗∗^	0.165
	(0.155)	(0.131)	(0.326)	(0.339)	(0.234)	(0.222)	(0.126)	(0.109)

Region FE	Yes	No	Yes	No	Yes	No	Yes	No
Observations	690	690	690	690	690	690	690	690

*Note:* Robust standard errors in parentheses.

^∗∗∗^ *p* < 0.01.

^∗∗^ *p* < 0.05.

^∗^ *p* < 0.1.

**TABLE 4:** Average impacts.

Variables	Direct effect	Indirect effect	Total effect
Stunting			
Precipitation	−0.334^∗∗^	−0.048^∗^	−0.381^∗∗^
	(0.135)	(0.027)	(0.152)
Aridity	0.288^∗∗∗^	−0.041^∗^	0.330^∗∗∗^
	(0.108)	(0.022)	(0.121)
Number of wet days	−0.017	−0.002	−0.019
	(0.061)	(0.009)	(0.070)
Daytime land surface temperature	0.135^∗∗^	0.019^∗^	0.155^∗∗^
	(0.057	(0.011)	(0.064)
Skilled health worker availability	−0.053^∗∗^	−0.008^∗^	−0.061^∗∗^
	(0.021)	(0.004)	(0.024)
Women’s education	−0.299^∗∗∗^	−0.043^∗∗^	−0.341^∗∗∗^
	(0.051)	(0.021)	(0.059)
Location (ref: rural)	−0.036^∗^	−0.005	−0.042^∗^
	(0.019)	(0.004)	(0.022)
Wasting			
Precipitation	0.096	0.082	0.178
	(0.081)	(0.066)	(0.145)
Aridity	−0.127^∗∗^	−0.108^∗∗^	−0.235^∗∗^
	(0.064)	(0.054)	(0.114)
Number of wet days	0.012	0.010	0.023
	(0.036)	(0.031)	(0.066)
Day time land surface temperature	0.003	0.003	0.006
	(0.033)	(0.028)	(0.061)
Skilled health worker availability	−0.001	−0.001	−0.001
	(0.012)	(0.010)	(0.023)
Women’s education	−0.074^∗∗^	−0.063^∗∗^	−0.137^∗∗^
	(0.030)	(0.028)	(0.055)
Location (ref: rural)	−0.002	−0.002	−0.005
	(0.011)	(0.009)	(0.020)
Undernutrition			
Precipitation	−0.367^∗∗∗^	−0.031	−0.398^∗∗∗^
	(0.132)	(0.020)	(0.142)
Aridity	0.219^∗∗^	0.018	0.237^∗∗^
	(0.105)	(0.013)	(0.113)
Number of wet days	0.014	0.001	0.015
	(0.060)	(0.005)	(0.818)
Day time land surface temperature	0.007	0.001	0.007
	(0.055)	(0.005)	(0.060)
Skilled health worker availability	−0.009	−0.001	−0.009
	(0.020)	(0.002)	(0.022)
Women’s education	−0.389^∗∗∗^	−0.033^∗^	−0.422^∗∗∗^
	(0.050)	(0.019)	(0.055)
Location (ref: rural)	−0.048^∗∗∗^	−0.004	−0.052^∗∗∗^
	(0.018)	(0.003)	(0.020)

*Note:* Standard errors in parentheses.

^∗∗∗^ *p* < 0.01.

^∗∗^ *p* < 0.05.

^∗^ *p* < 0.1.

**TABLE 5:** OLS regression results.

	(1)	(2)	(3)	(4)	(5)	(6)	(7)	(8)
Variables	Stunting	Stunting	Wasting	Wasting	Underweight	Underweight	Undernutrition	Undernutrition
Precipitation	−0.394^∗∗^	−0.574^∗∗∗^	0.261^∗∗∗^	0.344^∗∗∗^	0.195	0.065	−0.399^∗∗∗^	−0.462^∗∗∗^
	(0.154)	(0.104)	(0.088)	(0.063)	(0.126)	(0.091)	(0.136)	(0.098)

Aridity	0.342^∗∗∗^	0.446^∗∗∗^	−0.263^∗∗∗^	−0.310^∗∗∗^	−0.245^∗∗^	−0.127^∗^	0.245^∗∗^	0.280^∗∗∗^
	(0.122)	(0.084)	(0.070)	(0.050)	(0.100)	(0.072)	(0.108)	(0.078)

Number of wet days	−0.018	−0.012	0.001	−0.030	0.043	−0.003	0.014	0.018
	(0.060)	(0.056)	(0.040)	(0.035)	(0.058)	(0.050)	(0.062)	(0.054)

Daytime land surface temperature	0.151^∗∗^	0.054	−0.010	0.059	−0.027	0.046	0.012	−0.005
	(0.060)	(0.059)	(0.037)	(0.036)	(0.053)	(0.052)	(0.057)	(0.055)

Skilled health worker availability	−0.059^∗∗^	−0.010	0.015	0.013	0.012	0.052^∗∗∗^	−0.010	0.041^∗∗^
	(0.024)	(0.025)	(0.014)	(0.013)	(0.020)	(0.019)	(0.021)	(0.021)

Women’s education	−0.313^∗∗∗^	−0.341^∗∗∗^	−0.110^∗∗∗^	−0.133^∗∗∗^	−0.341^∗∗∗^	−0.379^∗∗∗^	−0.403^∗∗∗^	−0.469^∗∗∗^
	(0.053)	(0.059)	(0.033)	(0.033)	(0.048)	(0.047)	(0.052)	(0.051)

Location (ref: rural)	−0.035^∗^	−0.074^∗∗∗^	−0.011	−0.003	−0.039^∗∗^	−0.055^∗∗∗^	−0.048^∗∗^	−0.085^∗∗∗^
	(0.019)	(0.019)	(0.012)	(0.012)	(0.018)	(0.017)	(0.019)	(0.019)

Constant	0.779^∗^	1.403^∗∗∗^	−0.280	−0.628^∗∗∗^	0.208	0.188	1.497^∗∗∗^	1.611^∗∗∗^
	(0.430)	(0.371)	(0.250)	(0.211)	(0.361)	(0.306)	(0.388)	(0.329)

Region FE	Yes	No	Yes	No	Yes	No	Yes	No
Observations	690	690	690	690	690	690	690	690
R‐squared	0.276	0.185	0.326	0.250	0.345	0.263	0.356	0.278

*Note:* Robust standard errors in parentheses.

^∗∗∗^ *p* < 0.01.

^∗∗^ *p* < 0.05.

^∗^ *p* < 0.1.

Table 4 shows direct, indirect and total effects of the control variables on undernutrition in Ethiopia.

The effects of precipitation on stunting and undernutrition are negative and significant, while the aridity and day land surface temperature were positive and significant. In addition, direct, indirect and total effects of socioeconomic variables (skilled health workers, women’s education and location) on stunting, wasting and undernutrition were negative and significant. The negative spillover effects for these variables are less than the negative direct effect, highlighting a favourable decline in undernutrition, stunting and wasting for neighbouring clusters with high undernutrition. The number of wet days insignificantly affected undernutrition.

## 6. Discussion

Child undernutrition is a public health challenge, mostly affecting vulnerable populations, hence denying them an opportunity to live a healthy life. The adverse effects of child undernutrition are far‐reaching, crippling the whole country, not only individuals and households. Every child deserves a healthy life and well‐being without facing any challenges. This can be done through proper and balanced nutrients for children in the early years, which are critical for development. This is also considered a key strategy for long‐term health benefits for everyone. To contribute to this agenda, we focused on the geographical inequalities in stunting, wasting and underweight in Ethiopia.

We noted evidence of spatial disparities in stunting, wasting and underweight in Ethiopia, where different kebeles experienced different child undernutrition prevalence. Kebeles in the northern, northeast, southwest, central and western regions had a high stunting prevalence in 2019. In addition, some kebeles in the northern, northeast, southern and southeast of Ethiopia had a high proportion of children underweight, while those in eastern and some parts of northern and northeast regions were heavily affected by wasting in under‐five children. Children in the northern and northeast faced the triple burden of stunting, wasting and underweight. The results align with findings from existing literature, which also found spatial inequality in child undernutrition [5, 7, 15–17, 32]. These disparities have adverse effects on the education and labour outcomes for people living in different regions, hence influencing living standards and development.

Focusing on weighted variables’ coefficients, stunting, wasting and undernutrition spillover were positive and significant across clusters. There is evidence of positive spatial interdependence of undernutrition in Ethiopia. Changes in undernutrition in one cluster influence undernutrition in the neighbouring clusters. We found that undernutrition clusters geographically as clusters share risks and influence each other. This might be a result of shared environmental conditions, socioeconomic factors, healthcare services and cultural practices between clusters.

Focusing on climate change variables, we found that aridity, daytime land surface temperature and precipitation affect undernutrition among children under five in Ethiopia. Ahmed et al. [19], Chari [5] and Usman and Kopczewska [33] also found that climate variables influence stunting in Pakistan, Zimbabwe and Ethiopia, respectively. According to Ahmed et al. [19], climate change is linked to high temperatures, low precipitation and more arid areas, hence adversely affecting production, food and nutrition security. Over three‐quarters of the households in Ethiopia rely on subsistence farming, characterised by the overdependence on rain‐fed farming and keeping livestock with low crop production, hence less diversified foods, which negatively affects child undernutrition in the country [19].

The seemingly opposing effects of climate variables arise because of their influence on undernutrition through different pathways and time horizons. For example, increased precipitation improves agricultural productivity and reduces stunting, while increasing exposure to infectious diseases and environmental shocks that increase wasting in Ethiopia. On the contrary, aridity worsens long‐term food insecurity and stunting but may reduce short‐term health shocks and disease exposure, leading to lower levels of wasting. The results highlight the need to integrate interventions that address both dimensions of undernutrition. This can be done by investing in water, sanitation and hygiene infrastructure in wards that experience high levels of rainfall to mitigate disease‐related risks. Arid wards require sustained efforts to improve food security and resilience to chronic environmental stress. It is important to recognise the spatial and climatic heterogeneity within Ethiopia for designing effective and targeted nutrition policies.

Skilled health worker availability is also important in improving child nutrition in society, as indicated by [10, 27]. Having the necessary skills available at the facility significantly reduces child stunting by 0.059. This is because children in a kebele with more skilled health workers are more likely to receive good health, including nutritional advice, which helps them improve their nutritional status [10]. This aligns with Chari [5]’s findings in Zimbabwe. Moreover, women’s education is significantly associated with child nutrition [27]. Educated mothers give birth to children who are less stunted, wasted and underweight in Ethiopia, as these women are more empowered and financially capable of ensuring that their children have proper dietary uptake that improves their nutrition [10, 26]. The results from our analysis align with the results from [17, 26]. As noted in the literature, women who are highly educated are cognisant of their children’s nutritional status, and education and training in nutritional programmes are important in improving nutrition among children under five through social behavioural change.

Our analysis showed that a significant spatial distribution of undernutrition for children under the age of five exists in Ethiopia, which is important for prioritisation of regions grappling with undernutrition. We also identified significant contributors to child undernutrition that can be adjusted to alleviate the challenge. Our analysis also adds to the literature on spatial inequality in child undernutrition based on the lowest level of the subnational administrative boundaries’ data. Notwithstanding the crucial insights from the analysis, there are several limitations to be noted. EDHS lacks information on children’s food intake, which is important in showing whether the child has poor dietary uptake or not, as a contributor to child undernutrition. EDHS, like other survey data, is prone to selection, sampling, nonresponse and recall biases. Longitudinal data can be provided to analyse the causal effects of climate change on child undernutrition as well as investigate the intertemporal dynamics of child undernutrition in Ethiopia over time. This was not possible for our study due to the limited data on these variables in Ethiopia.

## 7. Conclusion

Child undernutrition remains a global challenge affecting poor and rural populations in most developing countries. Considering its adverse short‐ and long‐term effects on the vulnerable population, it is crucial to alleviate child undernutrition at the household and national levels. Most countries and nongovernmental organisations are rallying towards ensuring a healthy life for everyone, especially vulnerable populations (for example, children under five). We also contributed towards this important agenda by exploring the spatial disparities and factors affecting child undernutrition in Ethiopia. In that regard, we provided spatial analysis at the lowest administration level, which has not been done in previous studies. The results showed that the prevalence of stunting, wasting and underweight among under‐five children in Ethiopia is high. Spatial disparities in child undernutrition are present, with some parts of the country’s northern, northeast, southern and eastern regions experiencing a higher prevalence than other regions. Precipitation, aridity, daytime land surface temperature, skilled worker availability, women’s education and location significantly determine child undernutrition. Thus, policymakers need to design better educational and economic strategies to increase food security and dietary nutrition for children from poor and rural households, especially those in the northern, southern, northeast and some eastern parts of the country. Specifically, there is a need to ensure targeted nutrition programmes in arid regions, climate‐resilient agricultural interventions, expansion of maternal education programmes and strengthening rural health systems. This is important in ensuring the attainment of zero hunger and good health and well‐being.

## Funding

No funding was received for this manuscript.

## Conflicts of Interest

The authors declare no conflicts of interest.

## Data Availability

The data that support the findings of this study are available in the 2019 Ethiopia Mini Demographic and Health Survey at https://share.google/zpEldCzFimO6Ya6Le. These data were derived from the following resources available in the public domain: https://dhsprogram.com/pubs/pdf/FR363/FR363.pdf, https://dhsprogram.com/pubs/pdf/FR363/FR363.pdf.
